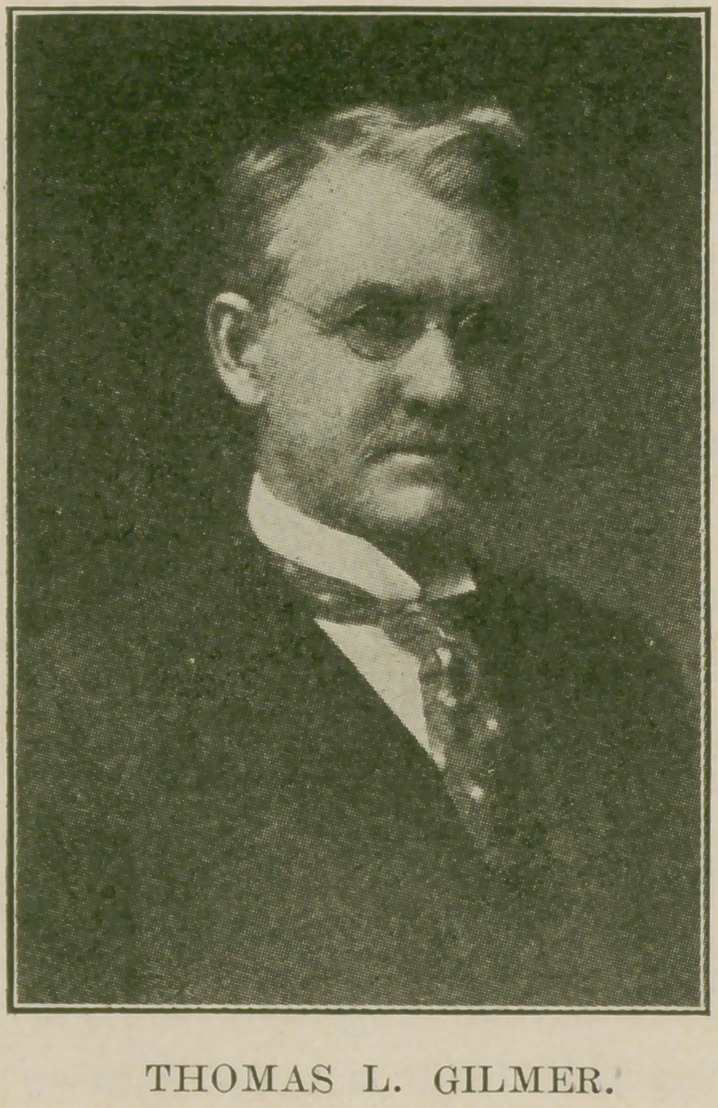# Chronic Oral Infections and Their Relation to Diseases in Other Parts*Read before Toronto Dental Society, 17th January, 1916, and published in *Oral Health* for February, 1916.

**Published:** 1916-04

**Authors:** Thomas L. Gilmer


### CHRONIC ORAL INFECTIONS AND THEIR RELATION TO DISEASES IN OTHER PARTS.*

THOMAS L. GILMER, M.D., D.D.S., SC.D.

The knowledge of focal infection in the mouth and jaws as a cause of systemic disturbances is by no means new. Many years before the advent of bacteriology, medical men often recognized the impossibility of curing patients having certain chronic disorders until their mouths were freed from infection. The prevailing method of treatment of disease dependent upon lesions of the gums and teeth at that time was the removal of the teeth. It was observed that after the mouth was freed from disease there frequently followed prompt recovery of patients from chronic physical disturbances, which before were incurable by other treatment.

THOMAS L. GILMER.

The association of the mouth with systemic disorders at that early day was the result of observation, and not from any scientific knowledge in regard to the subject. Bacteriology, as a science, had not then been demonstrated, and even after the development of bacteriology into a fixed science, many years elapsed before a close connection was made between cause and effect.

*Read before Toronto Dental Society, 17th January, 1916, and published in *Oral Health* for February, 1916.

In a series of articles by Miller in 1891, entitled "The Human Mouth as a Focus of Infection," he said (*Dental Cosmos,* September, 1891, page 689): "During the last few years the conviction has grown continually stronger among physicians, as well as dentists, that the human mouth as a gathering place and incubator of pathogenic germs performs a significant role in the production of varied disorders of the body, and that if many diseases whose origin is enveloped in mystery could be traced to their source, they would be found to have arisen in the oral cavity." These articles, written a quarter of a century ago, were prophetic of facts now demonstrable.

In 1904, Frank Allport, of Chicago, read a paper before the Chicago Dental Society, entitled, "The Relation of Odontology to Opthalmology and Otology," in which he quoted from various opthalmological and other medical journals, citing numerous cases of eye diseases which were secondary to jaw infections. (Chicago *Dental Review,* April, 1904.)

In 1906, Osler gave your own Hunter credit for having first called attention to oral infection as a cause of systemic disorders, and then made this statement (Osler *Practice of Medicine,* 6th edition, page 440): "Of the twenty cases of pernicious anemia which I had under observation in 1904, pyorrhea alveolaris was present in more than half." He further said: "Certain types of nephritis are also believed to be due to oral infection."

Hunter, of London, delivered an address at the opening session of McGill University Medical School, Montreal, October 3, 1910, entitled, "The Role of Sepsis and of Antisepsis in Medicine." (*The Lancet,* London, England, January 4, 1911.) In this address he cited numerous cases of systemic disorders which were directly traceable to oral sepsis, and criticized "American conservative dentistry" in severe terms. He also criticized medical men for looking everywhere except the right place—the mouth—for the cause of certain diseases. Some American dentists were highly indignant at this severe arraignment by Mr. Hunter, and retorted in a manner which, in the light of subsequent events, has made them appear in an unenviable light.

It was not until Billings, of Chicago, and others, in more recent years, systematically took up the subject and connected the clinical findings with scientific bacteriological demonstrations, that the true relationship existing between oral foci and systemic diseases were made manifest.

Billings associated with himself in this work Davis and Rosenow, bacteriologists, of Chicago, the latter now of the Mayo Foundation, in Rochester, Minnesota. The work of these men has created widespread interest in the subject and much good is resulting. Many valuable reports have been made by them and investigations are still going on, developing new and interesting facts.

Among their first work, diseased tonsils were subjected to bacteriological examinations, cultures made from the prevailing organisms isolated and animals inoculated. Joint, muscle, stomach, kidneys and heart lesions were demonstrated in these animals after death. The organisms, principally a streptococcus, regained from the animal lesions were found to be the same as those isolated from the diseased tonsils. Later it was found that abscessed teeth gave the same organisms, and these were found to produce on animals similar results to those taken from tonsils.

Billings says (*Journal of the American Medical Association,* May 1, 1915, page 1524): "Acute rheumatism has long been recognized as an infectious disease. The organism has been designated as Streptococcus rheumaticus, Diplococcus rheumaticus, or Micrococcus rheumaticus. With the phenomenal work done by Rosenow in connection with new methods of bacterial culture in our clinic, we practically always find the organisms in the exudate or in the tissues of the patient. We can then make a culture of it, can inject it into animals, producing the disease, and we can recover it from the animal again. Acute articular rheumatism is an infectious disease, due to a form of streptococcus; that streptococcus is specific in the production of the syndrome we call acute rheumatic fever, just as the pneumococcus is in producing ordinary lobar pneumonia. It has been proved by investigation that the organism in both acute and chronic rheumatism is hematogenous in its method of infection; the organisms passing through the smaller blood vessels of the tissues are caught by the endothelium of the vessel, producing cellular proliferation and obstruction of the blood vessel, so that in acute conditions one finds the blood vessel obstructed with minute hemorrhages at that point into the infected tissue.''

In 1911, Billings, Davis and myself, in a symposium in the Chicago Medical Society, presented papers on focal infections. I quote the following from my paper (Chronic Oral Infections. *Archives of Medicine,* April, 1912, vol. 9, pp. 499-504): ''Chronic alveolar abscess is so common that few go through life without one or more such suppurations. There are two forms of this disease: that which continually or periodically discharges either into the mouth, nose, maxillary sinus, or elsewhere, and the blind abscess which never discharges through a sinus, the pus being absorbed by the granulating walls of the abscess. The former, discharging pus and bacteria as indicated, can not but be prejudicial to health, and the latter, the blind abscess, offers greater danger. Its presence is generally unobserved by the patient and may be overlooked by the physician, even if the mouth is ocularly examined, since it may not present to the eye the clinical evidence of its presence. I think twenty-five per cent. a safe estimate of the percentage of jaws having suppurating cavities. That greater havoc is not wrought by such focuses of infection is due either to the fact that the dosage of bacteria and their poisons is usually insufficient, or to the fact that the normal individual becomes immune to the bacteria. Instances, however, are not lacking which demonstrate that such focuses of infection are instrumental in causing neuritis, neuroses and secondary infections of the eye, ear and other parts; therefore, when there are manifestations of diseases which may be dependent on some local focus of infection, the mouth and jaws, as well as other possible sources, should be examined to discover if they may not hold the key to the solution of the problem.

"The question which has not been answered, but must and will be, is: What is the bacteriology of chronic alveolar abscess? When this question and the question of susceptibility and immunity are answered, we can better understand the relation which these pathological cavities in the maxillae bear to lesions in the heart, kidneys, lungs, nerves and brain. Here are possibilities not to be ignored. Did similar abscesses exist in other bones of the body, their presence would demand immediate attention, both by the patient and the physician. Alveolar abscess is usually considered inconsequential, and when the attention of the profession has been called to it, it has generally excited little interest, and has been allowed to pass as an insignificant 'gum boil.'"

The etiology and pathology of alveolar abscess is so well known that its recitation in this paper would be superfluous. Its prevalence and dangers are now well recognized, and a more extended observation leads me to conclude that the above estimate of twenty-five per cent. for adults having chronic alveolar abscess is too low. In many instances these abscesses are not discoverable except by the aid of the radiograph, and in some instances the infected area is very small, but no matter how small the area involved, the danger may be quite serious.

In the quotation previously made from my paper on "Chronic Oral Infections," I said: "The question which has not, but must and will be answered, is, What is the bacteriology of chronic alveolar abscess?" At the meeting of the American Medical Association in 1914 (A Study of the Bacteriology of Alveolar Abscess and Infected Root Canals, Gilmer and Moody, *Journal of the American Medical Association,* December 5, 1914, Vol. 63, page 2023), Moody and I attempted to answer this question in a report of a short series of examinations made by us at St. Luke's Hospital Laboratory, and a similar study has since been made by Hartzel ("The Clinical Type of Arthritis Originating About the Teeth," *Journal of the American Medical Association,* 1915, LXV, 1093). We examined specimens from sixteen acute alveolar abscesses, eighteen subacute or chronic abscesses, and eight specimens from the root canals of abscessed teeth. In this series of forty-two cases we found the predominating organisms to be streptococci. We obtained many graded varieties from a hemolitic streptococcus with a wide zone of hemolysis in acute abscesses, to a streptococcus viridans in the chronic form. In one instance we found the streptococcus mucosus was the prevailing organism. After the report of this series, we made other examinations with similar results. We are now making a more extended study of chronic jaw abscesses with animal inoculation and passage. A partial preliminary report is appended below.

Unless the greatest care is exercised in the collecting of material for culture work the results will be valueless. In Moody's and my work the gums about the abscessed tooth and those adjoining are thoroughly cleansed with alcohol, a steril instrument is used to remove the tooth, and as it is removed it is not permitted to touch any part of the mouth. A sterile pipet drawn to a fine calibre and sealed with heat is used to collect the specimen, its large end being plugged with sterile cotton. The sealed end is broken off and flamed, and the material from the apical portion is sucked into the pipet and the opening sealed by fusing the glass. It then goes direct to the laboratory for culture.

Here is the preliminary report referred to above, made for me by my associate in this work, Dr. A. M. Moody, bacteriologist, St. Luke's Hospital. This study is being made for the purpose of determining, so far as possible, the effect on animals injected with strains of freshly isolated streptococci from chronic alveolar abscesses. In this work strains of streptococcus viridans, isolated from alveolar abscesses in fifteen patients suffering from various pathological conditions, have been injected into a total of forty-seven rabbits. A complete report of this work will be ready for publication in the early summer.

Since the results of these histological examinations are identical with those published in 1914 by Gilmer and Moody, it does not seem that a complete statement is necessary at this time. This much is true, that the streptococcus viridans in every instance is the predominating organism, and that in only one instance was the staphylococcus found, and then just an occasional colony was present.

Of the fifteen patients with chronic alveolar abscesses six had also pyorrhea; eight had rheumatism, one each acute gastric ulcer, neuritis, myorcarditis, mitral endocarditis, and nephritis. Rosenow's technique, in a large measure, has been followed in these studies. The exceptions are two, *i. e.* (1) The doses of streptococci have, in all instances, been less than two billion, and in most cases between one-half and one billion. These are approximate numbers. (2) The animals have been allowed to live a longer time after injection.

The gross pathological lesions present in the forty-seven rabbits are given below. The microscopical examinations of these have not as yet been completed, but in so far as these observations have progressed, the gross diagnoses have been confirmed. Following the table of Rosenow in the animals autopsied:

Appendicitis was present in .......... 2%
Hemorrhage of stomach ............. 40%

| | |
|---|---|
| Ulcer of stomach | 13% |
| Ulcer of duodenum | 2% |
| Hemorrhage or pus in gall bladder | 13% |
| Hemorrhage in pancreas | 15% |
| Hemorrhage into peritoneum | 5% |
| Arthritis and periostitis | 40% |
| Endocarditis | 28% |
| Pericarditis | 5% |
| Myocarditis | 5% |
| Nephritis | 30% |
| Hemorrhages or other lesions of the lungs | 10% |
| Hemorrhages into the skin | 2% |
| Tongue | 0% |
| Eye | 4% |
| Hemorrhages into jaw | 20% |

The hemorrhages into the jaw have not been previously described, except in a paper on experimental scurvy by Jackson and Moody before the American Association of Pathologists and Bacteriologists in St. Louis, April, 1915. These .hemorrhages occur beneath the periosteum of the lower jaw before the central incisors. Occasionally they occur on only one side, but may be present on both.

This series is too small to draw any definite conclusions. They, however, indicate a certain amount of selective localization for the streptococcus viridans isolated from chronic alveolar abscesses. To be more specific, these organisms produced gross evidences of muscle involvement in 60%, joint and bone, aside from the jaw, in 40%, stomach in 40%, kidney in 30%, and jaw in 20%.

We occasionally find what I have termed atipical alveolar abscesses, the lateral abscess of Black on the sides of the roots of teeth having live pulps.

Black believed that these abscesses were due to acute pyorrhea alveolaris attacks, the infection extending from the gingival border root-wise through a narrow channel on the side of the root.

Since Moody and I have found in twenty per cent. of our cases sub-periosteal hemorrhages in the jaws, I am inclined to believe that similar hemorrhages may be found in the peridental membrane. If hemorrhages are caused by the streptococcus in the periosteum, may it not cause a like condition in the peridental membrane as well?

Since the area involved in hemorrhage may later become abscessed, then if the peridental membrane participates in like hemorrhages we have a seeming scientific solution of the atipical alveolar abscess. As yet we have not examined the peridental membrane for hemorrhages, but intend to look for them in this organ.

Rosenows' work is the most interesting of all that has been done in connection with the subject of focal infections. Reports of much that he has done may be found in the *Journal of Infectious Diseases* and the *Journal of the American Medical Association* in the past three or four years. Rosenow says (*Journal of the American Medical Association,* May 1, 1915, page 1524): ''The demonstration of streptococci in the focus of infection at the time of an attack of appendicitis that has affinity for the appendix when injected intravenously into animals, it seems to me, is good evidence, together with all the other facts, that the growth of the organism in the throat or in the focus of infection is primary, and that the disease in the appendix is a result of this, not by the swallowing of bacteria, but by embolic infection, getting into the circulation and finding in the appendix a favorable spot for growth.''

He further says: ''We have heard much in regard to the importance of various foci of infection. The breaking of the continuity of surface, the epithelium of the skin, the breaking of the continuity of mucous membranes anywhere should be regarded as a serious matter. We know the relationship of slight abrasions to highly virulent streptococci infection. After demonstrating the presence of bacteria of low virulence in this type of infection in cases of cholecystitis and appendicitis, why not believe they are also important? The focus is not only the place of entrance, but also the infection atrium. The transmutation of streptococci has been established. In one instance there is an affinity for joints, in another for the appendix, and in still others for the stomach and the gall-bladder. These types of streptococci are so much alike in their cultural characteristics and morphology that it is difficult to differentiate them; but when injected as isolated, they are different in their actions in animals.''

The radiograph is essential to the best understanding of the condition of the jaws. The faradic current, as recommended by Prinz, is the best method of definitely determining the life of the tooth pulp, provided it is not too much enveloped by gold or other metals. To radiograph teeth having live pulps, in seeking out jaw abscesses, is an unnecessary expense to which to subject a patient, and the extent of pyorrhea pockets may usually be determined very accurately by the use of delicate steel probes. All teeth having dead pulps, which promise any hope of being saved, should be radiographed for the purpose of determining the presence or the absence of root fillings, the extent of the abscessed area, if present, and the extent of the destruction of the peridental membrane. If the apical area involved is inconsequential, then treatment may be undertaken through the pulp canal by medication. If the apical portion of the root is denuded of its normal investment, even to a slight degree, then that part denuded is dead tissue and must remain such, since the peridental membrane is an organ of specialized tissue, which when once destroyed is never reformed, leaving the denuded portion of cementum and dentine permanently necrosed. Unlike necrosed bone, the necrosed end of a root can not be exfoliated. If too great an extent of the root be denuded of its peridental membrane to indicate treatment by medication through the root canal, then the necrosed part may be resected, the abscess curetted, and the usefulness of the tooth, in a majority of cases, be preserved. Unfortunately, this treatment is not equally applicable to all teeth. Resection is particularly applicable to the upper incisors, cuspids and bicuspids; less so to the corresponding lower teeth, rarely to the upper molars, and practically never to the lower molars, owing to the anatomical relations.

If roots to be resected are infected or imperfectly filled, then these conditions should be corrected before the operation. All teeth whose peridental membrane is largely destroyed should be extracted, as no treatment can cure such teeth.

As before said, any apical abscess, it matters not how small, is a source of danger. So long as vitality is high, the product of a small abscess may do no visible harm, but a slight trauma or a lowered vitality may change seeming immunity into susceptibility to even small dosages of bacteria, or their toxins, with secondary manifestations resulting, often of a very serious nature. If this is true, it is our duty to keep close watch over our patients and eradicate every focus of infection found in the mouth.

With knowledge of Rosenow's work on "Elective Localization of Streptococci," we can see the possibilities of the danger of these streptococcus infections of the jaws, and since the mouth is a part of the human anatomy, for which the dentist is especially responsible, a grave duty rests upon him. So far as possible he should prevent alveolar abscess and pyorrhea alveolaris, but if these diseases are present they should be eradicated, even if it necessitates the removal of the teeth involved. Teeth are valuable, but life and health are paramount.

I am skeptical about the permanent cure of pyorrhea alveolaris after deep pockets have been formed. If the pockets are not so deep, but that cutting away of the gums overlying will eliminate the pockets, we may, in some instances, by the combinations of this with other treatment, preserve the teeth without jeopardizing the patient's health or life. I would emphasize the importance of completely eradicating the pus pockets, otherwise there can be no hope for a condition which is safe.

The radiograph is well recognized as an important aid to diagnosis in chronic jaw infections, but unless the pictures are skillfully made and properly interpreted, one may be led astray by them. The angle of the exposure of the film, in its relation to the light and the relation of the film to the tooth, are both important factors. The buccal roots of the upper molars, in some instances, appear to be within the maxillary sinus, because the light was superimposed to the floor of the antrum. Indeed, in many instances, it is difficult to make a picture of some of these teeth which will afford accurate information as to the condition of the apices of the roots. One should always remember that radiographs are simply shadow pictures, therefore show only the apical, mesial and distal parts of the roots.

A few of many similar clinical cases which have come under my observation will be cited later, which seem to indicate very clearly that mouth foci are instrumental in causing systemic disorders, since on the removal of the original foci of infection, the patients have fully recovered. It must not, however, be expected that all patients with arthritis deformans and with other systemic disorders of long standing will be cured when the original foci are removed, or that there will be *immediate* cure on their removal, as time and other treatment is often necessary.

I think it may be stated with considerable accuracy that the more chronic the condition, the greater the time necessary for a cure after eradicating the focus of infection; and also that in a majority of those cases which do not yield to the removal of the focal cause the condition has existed so long that changes have taken place in the organ or parts involved, which make it impossible for such organ or part to regain its normal state.

Vaccines have been quite extensively used in the treatment of various diseases, and in some instances marked improvement has been observed. No hope for help from vaccines should be expected, however, until the focus of infection has been removed, and even then the vaccines employed must be the right strain, otherwise no good should be anticipated, and even harm may be done by their use. The use of stock vaccines is only a guess that the right strain will be included among the various strains employed. Such vaccines are not unlike "shot-gun" prescriptions, in which a number of drugs are combined with the hope that some one of the ingredients may be the desired one needed to effect a cure.

Billings says (*Journal of the American Medical Association,* May 1, 1915, page 1524): "For acute rheumatism vaccines have been used. If we are going to follow any principle in treatment of vaccination, we must stop and ask what these vaccines are expected to do. In acute infectious disorders, vaccines have not proved efficacious. When the living organisms in the body do not excite enough antidotes, it is not reasonable to inject more dead bacteria into the tissue to excite the defenses. Phylacogen or rheumophylacogen preparations are being used all over the country for rheumatism. In the advertisement of a certain pharmaceutic firm there is a report of 15,000 cases of rheumatism in which recovery ensued in 12,000 with the use of rheumophylacogen. I have been practicing medicine for thirty-five years, and I have not seen patients die from acute rheumatism *per se.* I have seen them die from the sequelae, that is, of heart conditions, later in life, etc. My professional friends know that I have not a good opinion of these preparations."

Relative to stock vaccines, Rosenow says (*Journal of the American Medical Association,* May 1, 1915, page 1524): "It is absurd to hope to get good results from streptococcus vaccines bought at the drug store and using them for these various diseases. What good will streptococcus vaccines, manufactured by various commercial laboratories, do in each specific case? A vaccine should be developed and given for each specific disease. Only autogenous vaccines should be used. In order to have autogenous vaccines, the micro-organisms must be proved; then you can proceed to treat the case intelligently."

A few cases which have come under my observation may serve to illustrate the baneful effects of focal infections of the mouth and the remedy which brought about a cure.

Mr. A., aged 76, had an attack of rheumatism of the ankle and joints of the foot. He came to my office by the aid of crutches. I found two teeth badly abscessed. These teeth were removed and good drainage secured. One week later he was able to walk without crutches. He has remained well after a period of seven months. The rapidity of this recovery is unusual, and a like rapid recovery should not be generally expected.

Mrs. R., aged 70, had had sciatica for a year. The ordinary treatment for such cases was not helpful. She had pyorrhea about most of her teeth. The pyorrhea pockets were so extensive that extraction of the teeth was indicated. Her sciatica was rather worse following the removal of the teeth, and it was not until six months had passed that she was free from pain. One year has elapsed since the extraction of the teeth, and for half of this time she has had no pain in the sciatic region.

Mrs. B., aged 60, had arthritis and neuritis extending over a period of two years. No focus could be discovered other than in the mouth. Had many crowns and bridges. Mechanically, this work was well done. Abscesses at roots of several teeth, gingivitis about all crowns. Pockets between some of the molars. Roots had not been very successfully filled. Teeth were removed. Condition did not improve for six weeks, then gradual improvement for six months, when patient was freed from all pain and discomfort.

Mr. S., aged 26, referred to my clinic for surgical treatment of large abscess in the upper jaw resulting from infected tooth. Patient had temperature of 100 degrees, was anaemic, skin pale, eyes dull; had not felt well for several months. Removed teeth and cleaned out large abscess in the bicuspid region. One week later patient much improved in appearance, temperature normal, color better. Two weeks later he was seemingly completely recovered.

Mr. K., attorney. Health steadily failing for five years. Was pale and anaemic, "extremely nervous and easily upset." Digestion poor. Might be considered a neuresthenic. Physician could find no focus of infection outside of the mouth, and sent patient to me for oral examination. My examination discovered deep pyorrhea pockets about several teeth and abscesses about the apices of others. One of the abscesses extended from cuspid to cuspid on upper jaw. Removed abscessed teeth and curetted abscess cavities. Extracted the worst pyorrhea teeth and treated those less affected. Improvement apparent in a short time. In three months was fully restored to health, and still remains so. Gained much flesh, and is no longer dyspeptic or "nervous."

It must not be forgotten that the primary focus of an alveolar abscess may at times be the tonsils, joint, or other part, and the jaw infection secondary to these. Sometimes an abscess forms at the end of a root, which has no relation to the mouth. In such cases the infection must be secondary, the organisms coming through the blood streams or lymphatics from a primary focus elsewhere.

Since dentists now know that mouth foci of infection sooner or later may cause secondary manifestations of more or less significance in other parts, what will their attitude be toward the subject? Will they continue the retention of loose, incurable, pyorrhea-infected teeth? Will they continue the use of badly infected roots as piers for bridges? Will they ruthlessly destroy pulps in sound teeth that bridges may be inserted, knowing the difficulty of perfectly filling most roots, and the impossibility of filling some? Will they ignore the dangers from incurable abscessed teeth and retain them indefinitely in the jaws? Will they continue to set bridges which are unsanitary? These are pertinent questions for us all.

## Figures and Tables

**Figure f1:**